# Characterising the classes of children and young people with mental health concerns based on reported service contact

**DOI:** 10.1002/jcv2.70014

**Published:** 2025-06-09

**Authors:** Frances Mathews, Chris Playford, Obioha C. Ukoumunne, Tamsin J. Ford, Tamsin Newlove‐Delgado

**Affiliations:** ^1^ University of Exeter Medical School Exeter Devon UK; ^2^ Sociology, Philosophy and Anthropology University of Exeter Exeter Devon UK; ^3^ NIHR Applied Research Collaboration South West Peninsula Department of Health and Community Sciences Faculty of Health and Life Sciences University of Exeter Exeter Devon UK; ^4^ School of Clinical Medicine Department of Psychiatry University of Cambridge Cambridge UK

**Keywords:** children, latent class analysis, mental health, young people

## Abstract

**Background:**

Exploring the similarities and differences of mental health‐based service contact behaviours for children and young people (CYP) and associated characteristics will allow for distinct analysis of identified groups, and inform both current support pathways alongside more focussed targeted intervention strategies.

**Methods:**

Using data from the Mental Health of CYP in England Survey, 2017, we fitted latent class analysis models to identify classes of CYP based on the type of service contact they received. Analysis was stratified by educational stage (aged 5–10, 11–16 and 17–19 years) owing to different help‐seeking pathways.

**Results:**

For each educational stage, the four‐class model was the best fit. Latent classes for children aged 5–10 years included, No Services, Community Services, Nonmedical Services, Contact all services. Children and young people reported different patterns of class membership by gender and ethnic group. Similar latent classes were identified for YP aged 11–16 years including: No Services, Nonmedical Services, Community Services, and Contact all services, however, stronger patterns of contact were found for nonmedical compared to community services. For those aged 17–19 years, classes included: No Services, Nonmedical Services, Specialised Services and Community and Health Services. Young people in the Specialist Service class had higher probabilities of being white/other compared to Black/Asian/Mixed/Other.

**Conclusion:**

CYP show different patterns of service contact across educational stages, with gender and ethnic disparities. Our findings could inform models of help, and support those designing and commissioning services to refocus and review where funding is best placed.

**Key Points**

- Support services for child and adolescent mental health are overwhelmed. Understanding help‐seeking clusters that form is required to effectively support those in need.
- Classes of help‐seeking clusters form differently by educational age.
- Demographic, social and diagnostic characteristics are different across the classes.
- Knowing how service use differs for children and young people (CYP) informs those funding and working in community and specialist mental health‐based services to better support key figures involved with their help‐seeking.

## INTRODUCTION

A recent systematic review of childhood mental disorders estimated that one in eight CYP in high‐income countries experience disorder level impairment requiring treatment (Barican et al., 2022). Seeking help for mental health concerns prevents the exacerbation of difficulties (Green et al., 2005). However, provision and availability of mental health services struggle with resources. A recent report estimated that globally governments spend on average just over 2% of their health budgets on mental health (World Mental Health Report (WMHR), 2022). Service availability is therefore likely highly inconsistent, and pathways to accessing support liable to differ. Indeed, McDonald, systematic review (2018) reports CYP pathways to mental health support as complex, understudied and needing greater understanding of help‐seeking behaviours.

A recent secondary data analysis of CYP in England described the prevalence of mental health reported service contact for those with and without a Diagnostic and Statistical Manual of Mental Disorders, Fifth Edition (DSM‐5) diagnosis (Mathews, Ford, White, Ukoumunne, & Newlove‐Delgado, 2024). The current paper extends this work to characterise classes of CYP based on patterns of contact with informal and professional services including schools, general practitioner, specialist mental health services, for a mental health concern. Understanding the demand placed on different service types alone does not allow us to identify if multiple services are utilised on CYP's journey to accessing help. A more nuanced understanding of constellations of service use can underpin measures to increase accessibility and enable individual services to maximise a joined‐up approach which results in better services for CYP as well as reducing costs (Hutchings & Elen Williams, 2014).

Exploration of access to mental health services, including barriers and facilitators, aim to increase awareness and promote adaptation of existing frameworks at an organisational level (Gondek et al., 2017; McDonald, 2018; Vusio et al., 2020). Targeted interventions focussing on behavioural activation also demonstrate the need to encourage CYP directional help‐seeking towards specific services depending on need (Aguirre Velasco et al., 2020). The recent Mental Health of CYP in England (MHCYP) survey report in 2022 found over 76% of parents of children aged 7–16 years report seeking help for a mental health concern from informal, professional educational and health services (Newlove‐Delgado et al., 2022). In addition, current literature suggests YP favour informal sources of support, which can influence willingness to seek additional help (Lynch et al., 2023). This is particularly important when seeking to support those from different ethnic and cultural backgrounds, particularly YP who are establishing their own understanding and practices around mental health (Coelho et al., 2022). Children and young people have also been shown to solicit advice from a variety of professional and informal services, but we currently do not know which CYP are more likely to reach out to the different services (Mathews, Benham‐Clarke, et al., 2024; McDonald, 2018).

We want to explore the ‘make‐up’ of the classes of informal and professional services that parents of CYP and YP report contact with when they need help with mental health difficulties. Uncovering these hidden and distinct groups patterns of service contact will help service providers, researchers and policy makers to and identify which groups are underserved and understand which services are predominantly contacted.

These groups will be further dissected to understand the demographic and diagnostic characteristics of CYP within these classes to identify need in current support pathways.

This study will address the following aims:

1. Use Latent class analysis (LCA) to identify classes of the types of services contacted for mental health concerns throughout the childhood years separated by three educational‐stage age groups: 5–10 years, 11–16 years and 17–19 years
2. Describe the basic demographic and diagnostic characteristics of CYP in the identified classes.

## MATERIALS AND METHODS

### Participants

This study was a secondary analysis of the Mental Health of CYP in England, 2017 survey data, a nationally representative probability sample drawn to represent the English population. From an invited 18,029 parents of CYP aged 2–19 years, 9117 completed an online or interview survey. Data were included from a total of 7654 children aged 5–16, and YP aged 17–19 years. Parents of children aged 5–16 reported on their child's mental health related service contacts over the previous year, and YP aged 17–19 self‐reported their mental health related service contact (Sadler et al., 2018). The survey received ethical approval from West London and Gene Therapy Advisory Committee Research Ethics Committee. Data access was approved by National Health Services (NHS) Data Access Request Service (DARS) (DARS‐NIC‐331532‐B5T0C). Further information on this dataset can be found at Sadler et al. (2018).

### Measures

Parents, CYP and if the family agreed, a teacher completed a standardised diagnostic assessment, the Development and Wellbeing Assessment (DAWBA) which was assessed by a team of clinical raters (including TND and TF) to assign psychiatric disorders according to the Diagnostic and Statistical Manual of Mental Disorders, Fifth Edition (DSM‐5) diagnoses (American Psychological Association, 2022; Goodman et al., 2000). For the analysis, the sample was split into age groups which align with educational stages (5–10 primary; 11–16 secondary; 17–19 work/higher education/not in higher education or training (not in education, employment or training (NEET))). Parents and YP aged 11–19 completed the Strength and Difficulties Questionnaire (SDQ) (Goodman, 1997) and 4‐band categorisation reported.

### Service contact variables

This secondary analysis used service contact variables from MHCYP 2017 (see Table 1), (Sadler et al., 2018).

**TABLE 1:** Question and response categories for reports of service contact.

Question	‘In the past year have you or <Name> been in contact with any of these people because of worries about < your/his/her > emotions, behaviour, concentration or difficulties in getting along with people?’
**Binary response categories (yes/no)**	***Example of role within category***
Informal services^a^	Someone in your family or a close friend, telephone helpline, Internet
Primary health care (primary care)	A GP, family doctor, health visitor, practice nurse or school nurse
Teachers and school staff (school support)	A tutor, head of year, headteacher or special educational needs coordinator
Education specialist	Educational psychologist, educational social worker or specialist teacher from outside school
Mental health specialist	A mental health nurse, psychiatrist, psychologist or counsellor
Physical health specialist (Child health)	A hospital or community paediatrician, or occupational therapist

^a^ Informal support amalgamated from these response categories for the purposes of this analysis owing to low numbers.

All service contact reports were binary (yes/no). Responses about social care and youth justice were excluded, as these contact types were so rare that their inclusion resulted in unstable models. Similarly, paediatric care (as listed in Child Health) rarely provide support for CYP over 16 years so were removed from the 17–19 analysis. Strength and Difficulties Questionnaire total difficulties self‐reports were only available for those aged 11–19 years (Goodman, 1997), as were social support scores. Ethnicity categories were White (White British/Other), Black (Black/African/Caribbean/Black British), Asian (Asian/Asian British) and Mixed (Mixed/Multiple/Other). Participants who did not respond to the question on service contact were not included in the analysis (5–10 years *n* = 14; 11–16 years *n* = 23; 17–19 years *n* = 9). We present the basic demographic data including Gender, Ethnicity and DSM‐5 diagnostic categories. A range of additional characteristic variables are available in supplementary information, separated by additional demographic, within‐household and social characteristics.

Sample characteristics are shown in Table 2.

**TABLE 2:** Sample characteristics by age group showing frequency (%).

Variables	Age groups		
	5–10 years	11–16 years	17–19 years
**N**	3583	3098	927
**Total N**	7608		
**Demographic**			
Gender			
Female, *n* (%)	1772 (49.5)	1558 (50.3)	454 (49.0)
Male, *n* (%)	1811 (50.5)	1540 (49.7)	473 (51.0)
Ethnicity			
White, *n* (%)	2824 (78.8)	2475 (79.9)	754 (81.3)
Black, *n* (%)	151 (4.2)	131 (4.2)	Black/Asian/Mixed 173 (18.7)
Asian, *n* (%)	371 (10.4)	304 (9.8)	
Mixed, *n* (%)	236 (6.6)	187 (6.0)	
**‘Service contact’ item response indicators**			
Informal, *n* (%)	468 (13.1)	412 (13.3)	368 (39.7)
School support, *n* (%)	613 (17.1)	519 (16.8)	114 (12.3)
Mental health specialist, *n* (%)	95 (2.7)	167 (5.4)	73 (7.9)
Primary care, *n* (%)	251 (7.0)	221 (7.1)	85 (9.2)
Special education support, *n* (%)	164 (4.6)	159 (5.1)	25 (2.7)
Child health, *n* (%)	130 (3.6)	93 (3.0)	n/a
DSM‐V diagnosis, *n* (%)	426 (11.9)	495 (16.0)	178 (19.2)

### Latent class analysis

We used Stata version 17 to undertake all analyses (StataCorp, 2021). Survey sample weights were applied to produce population estimates, explained in the MHCYP 2017 survey design and methods report (Vizard et al., 2018). Latent class analysis was undertaken using the six binary ‘service contact’ item response indicators as manifest variables using the *gsem* Stata command. Data is stratified by educational ages based on existing literature acknowledging different patterns of help‐seeking among different age groups. This separation also provides for more nuanced findings to better target intervention and directional help‐seeking.

Aim 1: We fitted separate LCA between 2 and 5 models for each age group to identify classes of CYP based on reports of ‘service contact’ received. The model estimates two types of *prior* probabilities (Bartholomew et al., 2008): the percentage in the population that falls in each class (marginal probabilities) and the percentage of people in each class that has received each type of ‘service contact’ (conditional probabilities). Initially, a model was fitted with two classes and the number of classes was increased until the model with the best fit and interpretability was reached. For estimation, model identification and fit, all LCA models were fitted with 300 sets of start values (pre‐set by Stata) which were increased to 400 when convergence was not achieved. In order to identify the best model the information was assessed in two ways. The first approach considered a number of criteria: Akaike Information Criterion which balances the complexity of the model against the size of the sample; Bayesian information criterion (BIC) quantifies the level of efficiency the model has in predicting the data (demanding a low log‐likelihood); entropy, with a possible range from 0 to 1, where a value of 1 indicates perfect fit; the bootstrap likelihood ratio test (BLRT) which compares the fit of a model with a given number of classes to that with one fewer model ‐ a statistically significant result at the 5% level was used as the threshold to indicate that the model with the additional class is a better fit (see supplementary Table S1). The second approach required the estimated conditional probabilities to be interpretable (Melendez‐Torres et al., 2018).

Aim 2: To compare the demographic and diagnostic characteristics between the latent classes we reported *posterior* probabilities, the percentage of each class that falls into each category of those variables. Statistical tests were undertaken to quantify evidence that the posterior probabilities truly differed between the latent classes for each of the age groups. For YP aged 17–19 years, the additional social characteristic of current education/training/NEET is included as YP are no longer required to remain only in education.

## RESULTS

Table 2 shows participant sample characteristics.

### Aim 1: Identifying class membership

#### Children aged 5–10 years

Supplementary Table S1 provides the summary of model fit statistics for models with 2 to 5 classes. Models of more than 4 classes were hard to interpret because they included more than one class with different distributions of the reports of ‘no services’. The 4‐class model was clearly interpretable based on the conditional probabilities; the BIC statistic shows it was most parsimonious, retains high entropy, and the BLRT confirms the model to be a marked improvement on the 3‐class model. After reviewing the conditional probabilities, the classes were named (see supplementary Table S2).

**The ‘4‐Class’ Model:** Class 1, interpreted as ***No Services***, was the largest, comprised of 84.9% of children. The majority in this class reported no use of each of Informal, Primary Care, School Support, Education Specialist, Mental Health Specialist or Child Health Services. Class 2 consisted of those who reported ***Community Services*** was comprised of 6.9% of children, characterised by majority contact with Informal, School Staff and Primary Care. Class 3, interpreted as ***Nonmedical Services*** comprised of 6.7% of children), was characterised by Informal and School Staff contact. This class comprised 6.7% of children. Finally, Class 4 ***Contact All Services***, comprised of 1.5% of CYP, was characterised by contact across all indicator services.

#### Young people aged 11–16 years

Supplementary Table S1 provides the summary of model fit statistics for models with 2 to 5 classes. Despite the 3‐class model being equally as interpretable to the 4‐class model, the 4‐class model had better model fit statistics, including high entropy, and was selected on this basis. After reviewing the conditional probabilities, the classes were named (see supplementary Table S5).

Supplementary Table S5 shows the latent class prior probabilities for the selected **‘4‐class’ model.** Class 1, interpreted as ***No Services*** was comprised of 78.5% of YP, and characterised by little use of each of Informal, Primary Care, School Support, Education Specialist, Mental Health Specialist or Child Health Services. Class 2, named ***Nonmedical Services*** was comprised of 13.1% of YP, and characterised by Informal and School Support contact. Class 3, named ***Community Services*** (6.9% of YP), was characterised by majority Informal, School Support and Primary Care contact. Class 4, ***Contact All Services***, was the smallest class, comprised of 1.6% of YP. It was characterised by contact across all service indicators.

#### Young people aged 17–19 years

Table 3 reports the fit statistics for models with 2 to 5 latent classes. The 3‐class model was hard to interpret, showing different distributions of ‘no services’ in two of the three classes, making them indistinguishable from one another. The 4‐class model was most parsimonious based on the BIC statistic, was clearly interpretable, had high entropy and the BLRT confirmed the model to be a better fit than the 3‐class model. While the 5‐class model showed improved entropy, it did not improve understanding as more than one class could be characterised as ‘no contact’. After reviewing the conditional probabilities, the classes of the 4‐class model were named (see supplementary Table S8).

The ‘4‐Class’ Model: Class 1, ***No Services***, was comprised of 71.1% of the 17–19 year olds. Most had no contact with any services. *Class 2*, ***Nonmedical Services*** (comprised of 17.6% of 17–19 year olds), characterised by majority contact with Informal and School Support. Class 3, ***Specialised Services***, accounted for 7.6% of 17–19 year olds, and was characterised by Informal, Mental Health Specialist and Primary Care contacts. Class 4, ***Community and Health Services***, comprised of 3.7% the population, and was characterised by Informal, School Support, Mental Health and Primary Care contacts.

### Aim 2: Demographic, social, symptom and diagnostic characteristics of the latent classes

The relationship of demographic, social and diagnostic characteristics with class membership was examined for the 4‐class model for each age group.

#### Children aged 5–10 years

The percentages of people in each class that fall into each category of the characteristics variables (posterior probabilities) are reported in Table 3 for those aged 5–10. Supplementary Table S3 shows the percentages for additional characteristics not reported in Table 3. Supplementary Table S4 shows the distribution of those assigned to each class by socio‐demographic group.

There were statistically significant differences between latent classes for all reported characteristics (*p* < 0.05).

**TABLE 3:** Posterior probabilities (%) of characteristics for children aged 5–10 years in the 4‐class model.

Covariate	Class 1	Class 2	Class 3	Class 4
	*No services*	*Community Services*	*Nonmedical Services*	*Contact all services*
**Demographic characteristics**				
	%	%	%	%
**Gender** ^a^				
*Female*	50.9	40.3	43.8	29.7
*Male*	49.1	59.7	56.2	70.3
**Ethnicity** ^a^				
*White*	77.0	86.7	92.7	89.1
*Black*	4.7	2.4	0.9	0.0
*Asian*	11.4	5.7	2.2	6.3
*Mixed*	6.9	5.2	4.3	4.7
**DSM‐V diagnoses characteristics**				
	%	%	%	%
Any DSM diagnosis^a^	6.4	53.6	30.9	73.4
Any anxiety diagnosis^a^	1.7	19.0	11.2	26.6
Any depressive diagnosis^a^	0.2	3.8	1.3	6.3
Any behavioural diagnosis^a^	1.4	27.0	15.9	37.5
Any ADHD diagnosis^a^	0.9	22.3	7.3	42.2
Any less common diagnosis^a^	3.1	23.7	10.3	39.1
More than one diagnosis^a^	0.8	26.1	11.2	45.3

^a^ = Chi2 test found significant difference between latent classes, *p* = < 0.005; *= categorised as representing 80% of the population.

**Demographic characteristics:** The percentage of males was higher in Community Services (59.7%), Nonmedical Services (56.2%) and Contact – All Services classes (70.3%) than in the No Services class. The percentage for Black, Asian and Mixed ethnic groups was higher in the No Services class than any other class. The opposite was seen in the White ethnic group.

**DSM‐V diagnoses characteristics:** The percentage of children with all types of DSM‐V diagnosis and those with more than one diagnosis was highest in the Contact ‐ All Services class, and lowest in the No Services class. The percentages were consistently higher in the Community Services class compared to the Nonmedical Services class across all DSM‐V diagnosis.

#### Young people aged 11–16 years

The relationship between socio‐demographic variables and class membership was examined for the 4‐class model for YP aged 11–16 years Table 4 reports the percentage of YP in each category of the socio‐demographic variables for each latent class. Supplementary Table S6 reports the same information for additional characteristics not reported in Table 4. Supplementary Table S7 shows the distribution of those assigned to each class by socio‐demographic group.

**TABLE 4:** Posterior probabilities (%) of characteristics for young people (YP) aged 11–16 years in the 4‐class model.

Covariate	Class 1 No services	Class 2 Nonmedical services	Class 3 Community services	Class 4 Contact all services
***Demographic characteristics***				
	%	%	%	%
**Gender**				
*Female*	50.7	46.6	51.9	49.1
*Male*	49.3	53.4	48.1	50.9
**Ethnicity** ^a^				
*White/Other*	77.0	91.0	95.7	94.3
*Black/African/Caribbean*	5.1	0.9	0.0	0.0
*Asian/Asian British*	11.3	3.6	3.2	0.0
*Mixed/Multiple/Other*	6.6	4.5	1.1	5.7
***DSM‐V diagnoses***				
	%	%	%	%
Any DSM diagnosis^a^	8.3	34.2	65.8	92.5
Any anxiety diagnosis^a^	3.7	13.8	35.8	62.3
Any depressive diagnosis^a^	1.4	6.9	11.8	30.2
Any behavioural diagnosis^a^	2.1	12.3	32.6	43.4
Any ADHD diagnosis^a^	1.0	8.4	18.7	32.1
Any less common diagnosis^a^	2.8	9.6	19.8	34.0
More than one diagnosis^a^	2.3	13.2	34.2	64.2

*Note:* Significant differences (*p* < 0.05) were found in all characteristics excluding: gender, *p* = 0.522.

^a^ = Chi2 test indicated significant difference between latent classes, *p* = < 0.005; *categorised as representing 80% of the population.

**Demographic characteristics:** The percentage of male and female showed an even split in all classes. The percentage of Black, Asian and Mixed ethnic groups was notably higher for the No Service class compared to any other class.

**DSM‐V diagnoses:** The percentage of YP with any DSM‐V diagnosis and more than one diagnosis was highest in the All Services class (92.5% and 64.2% respectively). Across all types of disorder, the percentage of YP in Contact All Services class was consistently higher than the Community Services class.

#### Young people aged 17–19 years

The relationship between socio‐demographic variables and class membership was examined for the 4‐class model; the findings are summarised in Table 5. Supplementary Table S9 reports the findings for additional characteristics not reported in Table 5. Supplementary Table S10 shows the proportion of those assigned to each class by socio‐demographic group.

**TABLE 5:** Posterior probabilities (%) of characteristics for young people (YP) aged 17–19 years in the 4‐class model.

Covariate	Class 1 *No Services*	Class 2 *Nonmedical Services*	Class 3 *Specialised Services*	Class 4 *Community and Health Services*
***Demographic characteristics***				
	%	%	%	%
**Gender** ^a^				
*Female*	45.8	57.1	62.9	64.3
*Male*	44.2	42.9	37.1	35.7
**Ethnicity**				
*White/Other White*	81.2	81.3	85.5	78.6
*Black/Asian/Mixed/Other*	18.8	18.7	14.5	21.4
***Social characteristics***				
	%	%	%	%
**Current education/training/employment** ^a^				
*FT education (up to A Level/equivalent)*	45.7	68.1	45.2	67.6
*FT education (above A Level/equivalent)*	22.0	20.9	12.9	18.9
*Employment/apprenticeship*	23.3	7.7	30.7	8.1
*Not in education or training*	9.1	3.3	11.3	5.4
***DSM‐V diagnoses***				
	%	%	%	%
Any DSM diagnosis^a^	13.2	33.0	51.6	45.2
Any anxiety diagnosis^a^	8.9	23.1	35.5	33.3
Any depressive diagnosis^a^	3.1	6.6	19.4	19.1
Any behavioural diagnosis	0.8	1.1	1.6	0.0
Any ADHD diagnosis	1.2	4.4	1.6	0.0
Any less common diagnosis^a^	3.0	6.6	14.5	9.5
More than one diagnosis^a^	3.1	6.6	16.1	14.3

*Note:* Significant differences (*p* < 0.05) were found in all characteristics excluding: Ethnicity, *p* = 0.709; conduct disorders, *p* = 0.855; ADHD, *p* = 0.110.

^a^ Chi‐squared test indicated statistically significant difference between latent classes, *p* = < 0.005; *categorised as representing 80% of the population.

**Demographic characteristics:** Overall, the percentage of YP in Nonmedical Services, Specialist and Community and Health Services' classes was higher for females than males. Membership in the White ethnic group was higher in every class than any other ethnic group.

**Social characteristics:** The percentage of YP in full time education was highest in the Nonmedical Services (89.0%) and Community and Health Services (86.5%) classes. The percentage of YP in Employment/Apprenticeship and NEET was highest in the Specialist Services class (30.7% and 11.3% respectively) compared to any other class.

**DSM‐V diagnoses:** The percentage of more than half of YP with a DSM‐V diagnosis was highest in the Specialised Services class (51.6%) and just under half in the Community and Health Services class (45.2%). Thoe percentage of those with a behavioural or ADHD diagnosis were lowest in Specialised Services and Community and Health Services classes.

## DISCUSSION

To our knowledge, this study is the first to report classes of CYP's group membership according to reports of service contact for mental health concerns from the MHCYP 2017 survey (Sadler et al., 2018). Our findings illustrate the likely patterns of help‐seeking across the various educational stages and the significance of their core characteristic profiles. This research provides service planners and providers with a clear understanding of the proportional distribution of membership and level of difficulties.

### Similarities and differences in class membership by age

It is clear from our findings that despite having almost exactly the same ‘service contact’ item response indicators, class membership differs for each educational stage, with the exception that the ***No Services*** membership was highest in the class across all ages. This supports current research highlighting the need to ensure the most utilised services receive maximum funding and support so that access inequalities are recognised, and a range of tailored intervention activities are offered by educational institutions to meet their needs (Mansfield et al., 2023). Importantly, most of those in the ***No Services*** class did not meet criteria for a DSM‐V Diagnosis, so would not be expected to be in a service contact class.

All educational age groups had a ***Nonmedical Services*** class. This is further discussed in ‘Membership in Community and Nonmedical services’ below.

For both 5–10 and 11–16 years, the ***Contact ‐ All Services*** class had proportionally smallest overall membership, but highest proportion of CYP with one or more DSM‐V diagnosis. This indicates that most CYP with DSM‐V diagnosis have had contact with a wide range of specialist mental health services and support. However, it does not include all those with a DSM‐V disorder. Edbrooke‐Childs et al. (2020) found that children in mental health services to be four times more likely to drop out depending on the service that was provided, and two‐and‐a‐half times more likely to drop out based on the practitioner providing treatment. This compounds the need to provide tailored treatments to ensure CYP complete treatments and improve mental health difficulties.

In line with other literature, classes of contact derived from YP aged 17–19 years self‐reports was markedly different to the younger age group's parent reports, showing a strong preference for informal help‐seeking, particularly friends and family (Lynch et al., 2023; Pretorious et al., 2019; Radez et al., 2021). Our findings show informal support to be an integral part of help seeking as it features strongly within the ***Community, Nonmedical, Specialised Community*** and ***Community and Health Services'*** classes. Young people in employment or training, and those NEET report greater membership in the ***No Services*** class, and lower membership in services which include medical and specialist MH support. Existing data shows NEET YP as being almost two times more likely to have experienced a MH difficulty compared to non‐NEET youth, (Goldman‐Mellor et al.). Alongside evidence that remaining in education is protective of health, there is clear need to better support this group with access to services (Reuter et al., 2022).

### Membership in community and nonmedical services

An important insight into help‐seeking by parents of CYP is seen from the even membership split between ***Community Services*** and ***Nonmedical Services*** class for those aged 5–10. There are likely multiple factors behind preference for service contact and help‐seeking among different groups, and barriers including stigma (Hansen et al., 2021; Reardon et al., 2017). This membership pattern indicates the way in which we collectively think about and respond to mental based difficulties, particularly regarding internalising and externalising difficulties (Jacobs & Loades, 2016; Rice et al., 2021).

The existence of the ***Community Services and Nonmedical Services*** class for those aged 5–10 and 11–16 years echoes other research showing schools to be a well‐established source of support for YP (Green et al., 2005; Sadler et al., 2018). Class membership is higher for those with lower needs and fewer adverse experiences, which fits the aim of school‐based mental health teams to manage mild and moderate difficulties (Department of Health and Social Care (DoHSC), 2021; Green et al., 2005). Interestingly, class membership in ***Nonmedical Services*** increases over the age groups, highlighting this pathway by which YP increasingly access support. This may reflect preference for less medicalised professional services in response to mental health difficulties and also how difficulties are channelled in the light of the additional provision of school funded mental health hubs which YP are more likely to seek support from (Department for Education, 2021; Transforming Children and Young People's Mental Health Provision: a Green Paper, 2018). However, it is important to consider recent increases in YP who are being homeschooled or have poor attendance rates who require medical professionals support service access.

By comparison, membership for those with a DSM‐V diagnosis is higher in the ***Community Services*** class, and increases again for ***All Services*** class, recognising the need for a range of support and services, for example, prescribing.

### Ethnicity

Unsurprisingly, our findings showed class membership to be high in the ***No Services*** class for those in Mixed, Black and Asian ethnic groups compared to those of White ethnicity. This was the opposite for membership in all other classes. This mirrors existing long‐standing records of difficulties with access and engagement for child and adolescent based mental health services among minority ethnic groups, including calls to support parental perceptions and beliefs which can be influenced by a cultural based stigma associated with mental health disorders (Gronholm et al., 2015; Lu et al., 2021; Ruphrect‐Smith et al., 2023). Lu et al. (2021) systematic review of service access among racial/ethnic minority adolescents identified that school experiences can impact access to mental health support, calling for schools to have a positive relationship with specialist services as it influences uptake. With one in five CYP in the UK speaking a language other than English in their home, the need for linguistic support in referral, assessment and provision of accessible treatment is imperative for both CYP and parents attempting to access support (Department for Education, 2024; Howard et al., 2024).

Problems are also reported for minority groups within services. Ruphrect‐Smith et al (2023) found differences in treatment outcomes from youth aged 18–20 years who had previous experience of mental health services. Young people from Asian and Mixed Race ethnic groups reported lower improvement in mental health following treatment compared to those in the White ethnic group. This range of barriers experienced by ethnic minority groups seems to reflect high membership in the ***No Services*** class. Alvarez et al (2022) suggest the need to adopt a top‐down approach in mental health services to increase diversity among practitioners and improve awareness and accessible education to meet minority groups needs.

### Gender

Our findings also support the long‐reported pattern of increased recognition of predominantly behavioural‐based mental health difficulties in primary school age boys (Green et al., 2005; Jacobs & Loades, 2016; Mathews, Ford, White, Ukoumunne, & Newlove‐Delgado, 2024; Sadler et al., 2018). Jacobs and Loades (2016) note that while recognition is not solely associated with behaviour, GP's report increased concern for Primary school aged boys compared to girls with regards to mental health. It highlights the importance of recognition of the differences displayed in the same disorders, for example, moving from stereotypical understanding and presentation of ADHD behaviours in boys and girls (Martin & Hadwin, 2022; Young et al., 2020). One recent study identified adolescent females aged 16–18 years to be more likely than their male counterparts to identify mental health difficulties and access therapeutic intervention despite perceiving greater barriers with regards to cost and ‘time‐wasting’ (Haavik et al., 2019). Even despite this, we find males aged 17–19 years have proportionally lower membership in classes reporting contact with a variety of support and services. This is particularly important for YP who mainly source informal based support from both friends and family, and increasingly via social media and web‐based platforms (Pretorius et al., 2019).

### Strengths and limitations

This study has many strengths, in terms of a carefully selected probability sample and the use of validated measures. The dataset provides a wide variety of types of services that parents of children and YP may have had contact with regarding concerns for mental health, including both informal and professional services. To our knowledge, this is the first study to analyse the hidden clusters of service contact of a representative sample of YP with mental health concerns.

Interpretation of our findings must be considered within the study limitations which include the depth and availability of some category variables, including gender (split by male/female). Another limitation is the use of parent report for children aged 5–16 and self‐reports for those aged 17–19 years. We acknowledge that the sample size for those aged 17–19 years, while representative and proportionate to the range of ages as well as reaching far beyond the recommended minimal sample size, differs from the other age groups (Finch & Bronk, 2011). Owing to this, there is the possibility that increasing the number of responses may result in a difference in the interpretation of classes. Aside from being reliant on recall, parents of children may not be aware of contact with teachers and school staff which may skew the reports recorded and therefore impact membership, and YP may not be willing to divulge contact. We also lack data on the type of intervention and experiences, for example, assessment or treatment provided as a result of contact. The ***No Services*** class may include reports of YP who are in need of help and support but are not accessing or able to access help. Any of these limitations may have had an impact on class solutions and factors including parsimony.

### What this study means today

This study uses the most recent representative population data available on CYP mental health which includes both DSM‐V diagnosis and reports of service contact in England, from 2017. It is therefore of utmost importance to understand how these clusters may have changed as a result of the pandemic, particularly given the wide increase in inequalities, changes in school attendance, delays in obtaining specialist mental health support and backlog of referrals (Magadi & Magadi, 2022; Morris & Fisher, 2022). This is particularly important when looking at the structure of the current classes, as it will help direct mental health service providers to develop and strengthen positive and symbiotic relationships among one another to increase access, information and support, as well as maximising engagement among and between parents, students and the community (Lu et al., 2021; Haavik et al., 2019; Roche & Strobach, 2019). Enabling a more diverse practitioner workforce may also serve to facilitate across both mental health and primary care for those in under‐represented groups, (Alvarez et al., 2025; Department of Health and Social Care (DoHSC), 2021). Careful consideration must also be made for CYP who need access to services who are not in school settings. Finally, it highlights the importance of how services communicate across all age groups, and the importance of strong leadership particularly between the community and school staff in order to maximise engagement both among and between parents, students and the community (Roche & Strobach, 2019).

## CONCLUSION

Understanding how service use clusters form for CYP across the educational stages provides a useful insight for those policy makers and service providers who are funding and supporting services. Typical mental health related demographic disparities are clearly identified.

## AUTHOR CONTRIBUTIONS

**Frances Mathews:** Conceptualization; formal analysis; methodology; writing—original draft; writing—review and editing. **Chris Playford:** Formal analysis; methodology; writing—review and editing. **Obioha C Ukoumunne:** Methodology; writing—review and editing. **Tamsin J Ford:** Conceptualization; funding acquisition; writing—review and editing. **Tamsin Newlove‐Delgado:** Conceptualization; funding acquisition; supervision; writing—review and editing.

## CONFLICT OF INTEREST STATEMENT

FM, TND, OU and CP have no conflicts of interest to declare. Tamsin Ford's research group receives payment for research methods consultations from Place2Be, a third sector organisation that provides mental health training and consultation to schools in the UK.

## ETHICAL CONSIDERATIONS

Ethical approval for secondary analysis from University of Exeter College of Medicine and Health Research Ethics Committee (Nov20/D/270) with the original survey obtaining ethical approval from West London and GTAC Research Ethics Committee.

## Supporting information

Supporting Information S1

## Data Availability

Approval for access to the MHYCP data obtained through the UK Data Service Data Access Request Service (DARS‐NIC‐424336‐T7K7T).
