# Supplementary material for: Characterising the classes of children and young people with mental health concerns based on reported service contact
Source: JCPP Adv. 2025 Jun 9;6(1):e70014. doi: 10.1002/jcv2.70014 (PMC12973121; doi:10.1002/jcv2.70014)
Supplement: Supplementary file 1 — Supporting Information S1 [file JCV2-6-e70014-s001.docx]

**Supplementary Information**

**Table S1: Model fit statistics for children and young people aged 5-10 years, 11-16 years and 17-19 years**

| **Age** | **Model** | **AIC** | **BIC** | **Scaled relative Entropy** | **BLRT (unweighted)** | **p-value** |
| --- | --- | --- | --- | --- | --- | --- |
| **5-10 years** | **2 class** | 8863.01 | 8943.40 | 0.89 | *-* | - |
|  | **3-class** | 8710.01 | 8833.69 | 0.84 | Comparing:  3 class to 4 class | 0.01 |
|  | **4-class** | **8670.96** | **8831.75** | **0.80** | **Comparing:**  **4 class to 5 class** | **0.02** |
|  | **5-class** | 8722.00 | 8888.96 | 0.82 | *-* | *-* |
|  |  |  |  |  |  |  |
| **11-16 years** | **2 class** | 8191.906 | 8270.406 | 0.89 | - | *-* |
|  | **3 class** | 8066.765 | 8187.535 | 0.82 | Comparing:  3 class to 4 class | 0.01 |
|  | **4 class** | **8046.809** | **8191.733** | **0.85** | **Comparing:**  **4 class to 5 class** | **0.42** |
|  | **5 class** | 8061.979 | 8261.250 | 0.79 | *-* | *-* |
|  |  |  |  |  |  |  |
| **17-19 years** | **2 class** | 2924.007 | 2977.158 | 0.72 | - | *-* |
|  | **3 class** | 2921.497 | 3003.640 | 0.71 | Comparing:  3 class to 4 class | 0.08 |
|  | **4 class** | **2906.404** | **3003.043** | **0.79** | **Comparing:**  **4 class to 5 class** | **0.07** |
|  | **5 class** | 2912.386 | 3023.521 | 0.82 | *-* | *-* |

**Table S2: Model 4: latent class probability of ‘yes’ to contact by item for children aged 5-10 years**

| **Indicators** | **Prevalence in sample** | **Class 1:**  ***No Services*** | **Class 2:**  ***Community Services*** | **Class 3:**  ***Nonmedical Services*** | **Class 4:**  ***Contact all Services*** |
| --- | --- | --- | --- | --- | --- |
|  | **%** | **%** | **%** | **%** | **%** |
| **Prior Probability of membership** |  | 84.9 | 6.9 | 6.7 | 1.5 |
| **Item** | **Probabilities** | | | | |
| **Informal Help** | 13.1 | 0.04 | 0.43 | 0.74 | 0.89 |
| **School support** | 17.1 | 0.05 | 0.68 | 1.00 | 0.97 |
| **Mental Health Specialist** | 2.7 | <0.01 | 0.11 | 0.04 | 0.77 |
| **Primary Care** | 7.0 | <0.01 | 0.63 | 0.16 | 0.80 |
| **Educational support** | 4.6 | <0.01 | 0.25 | 0.14 | 1.00 |
| **Child Health** | 3.6 | <0.01 | 0.36 | <0.01 | 0.72 |

**Table S3:** **Proportions of class membership posterior probabilities (%) by additional characteristics: children aged 5-10 years**

| **Covariate** | **Class 1** | **Class 2** | **Class 3** | **Class 4** |  |
| --- | --- | --- | --- | --- | --- |
|  | ***No Services*** | ***Community***  ***Services*** | ***Nonmedical Services*** | ***Contact All Services*** |  |
| ***Demographic characteristics*** | | | | |  |
|  | **%** | **%** | **%** | **%** |  |
| **Tenure^1^** |  |  |  |  |  |
| *Own* | 58.5 | 43.6 | 55.8 | 39.1 |  |
| *Private rented* | 20.6 | 24.2 | 22.3 | 28.1 |  |
| *Social rented* | 21.0 | 32.2 | 21.9 | 32.8 |  |
| **Income^1^** |  |  |  |  |  |
| *Highest tertile* | 35.0 | 23.9 | 34.7 | 18.8 |  |
| *Middle tertile* | 34.1 | 32.6 | 39.0 | 35.4 |  |
| *Lowest tertile* | 30.9 | 43.5 | 26.3 | 45.8 |  |
| **Region^1^** |  |  |  |  |  |
| *London* | 13.2 | 6.2 | 6.9 | 4.7 |  |
| *South of England* | 23.7 | 24.2 | 30.0 | 34.4 |  |
| *North of England* | 31.1 | 31.3 | 27.5 | 28.1 |  |
| *Midlands and East of England* | 31.9 | 38.4 | 35.6 | 32.8 |  |
| ***Within-household characteristics*** | | | | |  |
|  | **%** | **%** | **%** | **%** |  |
| **Parent Marital Status^1^** |  |  |  |  |  |
| *Married* | 65.5 | 53.1 | 57.1 | 48.4 |  |
| *Cohabiting* | 15.4 | 16.6 | 16.7 | 20.3 |  |
| *Lone parent – single* | 11.2 | 16.6 | 12.5 | 25.0 |  |
| *Lone Parent – previously married* | 7.9 | 13.7 | 13.7 | 6.3 |  |
|  |  |  |  |  |  |
| ***Social characteristics*** | | | | | |
|  | **%** | **%** | **%** | **%** |  |
| Stressful life events^1^ |  |  |  |  |  |
| *None* | 60.2 | 37.0 | 37.3 | 32.1 |  |
| *One* | 26.9 | 36.0 | 37.3 | 34.0 |  |
| *Two or more* | 12.9 | 27.0 | 25.3 | 34.0 |  |
|  |  |  |  |  |  |
| **Parent SDQ total difficulties scores^1^** |  |  |  |  |  |
| *Close to average** | 90.0 | 41.2 | 64.8 | 29.7 |  |
| *Slightly raised* | 5.1 | 14.2 | 11.7 | 6.3 |  |
| *High* | 2.3 | 11.9 | 10.7 | 14.1 |  |
| *Very high* | 2.6 | 32.7 | 13.3 | 50.0 |  |
| **Broken friendship/ relationship^1^** | 2.9 | 3.4 | 5.7 | 10.3 |  |
|  |  |  |  |  |  |
| **Child sent home for behaviour from primary school^1^** | 0.4 | 5.4 | 2.2 | 22.0 |  |
| **Child general health^1^** |  |  |  |  |  |
| *Very Good/Good* | 96.0 | 82.0 | 92.7 | 75.0 |  |
| *Poor* | 4.0 | 18.0 | 7.3 | 25.0 |  |
| **Child with SEND^1^** | 3.5 | 37.7 | 16.0 | 66.7 |  |
| **Looked after by Social Services at any point** | 0.7 | 1.4 | 1.7 | 3.8 |  |
| **Child is a carer^1^** | 1.9 | 0.5 | 3.9 | 0.0 |  |

**Table S4: Distribution of class membership posterior probabilities by all characteristics: children aged 5-10 years**

| Covariate | **Class 1**  ***No Services*** | **Class 2**  ***Community Services*** | | | **Class 3**  ***Nonmedical Services*** | | **Class 4**  ***Contact All Services*** | | |  |
| --- | --- | --- | --- | --- | --- | --- | --- | --- | --- | --- |
| ***Demographic characteristics*** | | | | | | | | |  |  |
|  | **%** | **%** | | | **%** | | **%** | | |  |
| **Gender^1^** |  |  | | |  | |  | | |  |
| *Female* | 88.4 | 4.8 | | | 5.7 | | 1.1 | | |  |
| *Male* | 83.4 | 6.9 | | | 7.2 | | 2.5 | | |  |
| **Ethnicity^1^** |  |  | | |  | |  | | |  |
| *White/Other* | 83.9 | 6.5 | | | 7.6 | | 2.0 | | |  |
| *Black/African/Caribbean* | 95.4 | 3.3 | | | 1.3 | | 0.0 | | |  |
| *Asian/Asian British* | 94.4 | 3.2 | | | 1.3 | | 1.1 | | |  |
| *Mixed/Multiple/Other* | 89.9 | 4.6 | | | 4.2 | | 1.3 | | |  |
| **Tenure^1^** |  |  | | |  | |  | | |  |
| *Own* | 88.0 | 4.5 | | | 6.3 | | 1.2 | | |  |
| *Private rented* | 84.0 | 6.8 | | | 6.9 | | 2.4 | | |  |
| *Social rented* | 82.2 | 8.7 | | | 6.5 | | 2.7 | | |  |
| **Income^1^** |  |  | | |  | |  | | |  |
| *Highest tertile* | 88.2 | 4.0 | | | 6.1 | | 0.8 | | |  |
| *Middle tertile* | 85.2 | 5.3 | | | 7.9 | | 1.5 | | |  |
| *Lowest tertile* | 84.2 | 7.8 | | | 5.8 | | 2.1 | | |  |
| **Region^1^** |  |  | | |  | |  | | |  |
| *London* | 92.7 | 3.0 | | | 3.6 | | 0.7 | | |  |
| *South of England* | 83.7 | 5.8 | | | 8.0 | | 2.5 | | |  |
| *North of England* | 86.7 | 6.0 | | | 5.8 | | 1.6 | | |  |
| *Midlands and East of England* | 84.2 | 6.9 | | | 7.1 | | 1.8 | | |  |
| ***Within-household characteristics*** | | | |  | |  |  |  |  |  |
|  | **%** | **%** | | | **%** | | **%** | | |  |
| **Parent Marital Status^1^** |  |  | | |  | |  | | |  |
| *Married* | 88.0 | 4.9 | | | 5.8 | | 1.4 | | |  |
| *Cohabiting* | 84.6 | 6.2 | | | 6.9 | | 2.3 | | |  |
| *Lone parent – single* | 81.2 | 8.2 | | | 6.8 | | 3.8 | | |  |
| *Lone Parent – previously married* | 78.9 | 9.4 | | | 10.4 | | 1.3 | | |  |
| **Parental mental health^1^** |  |  | | |  | |  | | |  |
| *Good* | 90.2 | 3.9 | | | 5.1 | | 0.8 | | |  |
| *Fair* | 83.6 | 7.1 | | | 8.2 | | 1.1 | | |  |
| *Poor* | 74.0 | 11.7 | | | 9.7 | | 4.6 | | |  |
| **Problematic Family functioning^1^** | 78.1 | 11.2 | | | 8.2 | | 2.4 | | |  |
| ***Social characteristics*** | | |  |  |  |  |  |  |  |  |
|  | **%** | **%** | | | **%** | | **%** | | |  |
| **Child general health^1^** |  |  | | |  | |  | | |  |
| *Very Good/Good* | 84.2 | 5.1 | | | 6.4 | | 1.4 | | |  |
| *Poor* | 63.4 | 19.6 | | | 8.8 | | 8.3 | | |  |
| **Child with SEND^1^** | 44.5 | 29.2 | | | 14.2 | | 12.0 | | |  |
| **Looked after by Social Services at any point** | 71.9 | 9.4 | | | 12.5 | | 6.3 | | |  |
| **Child is a carer^1^** | 85.5 | 1.5 | | | 13.0 | | 0.0 | | |  |
| **Stressful life events^1^** |  |  | | |  | |  | | |  |
| *None* | 91.1 | 3.8 | | | 4.3 | | 0.8 | | |  |
| *One* | 82.1 | 7.5 | | | 8.6 | | 1.8 | | |  |
| *Two or more* | 74.8 | 10.7 | | | 11.1 | | 3.4 | | |  |
| **Broken friendship/relationship^1^** | 75.2 | 6.0 | | | 13.7 | | 5.1 | | |  |
| **Child sent home for behaviour from primary school^1^** | 29.0 | 29.0 | | | 13.2 | | 29.0 | | |  |
| **Parent total difficulties scores^1^** |  |  | | |  | |  | | |  |
| *Close to average* | 91.5 | 2.9 | | | 5.0 | | 0.6 | | |  |
| *Slightly raised* | 72.2 | 13.9 | | | 12.0 | | 1.9 | | |  |
| *High* | 54.6 | 19.2 | | | 19.2 | | 6.9 | | |  |
| *Very high* | 38.0 | 32.4 | | | 14.6 | | 15.0 | | |  |
| ***DSM-V Diagnoses characteristics*** | | | | | | | |  |  |  |
|  | **%** | **%** | | | **%** | | **%** | | |  |
| **Any DSM diagnosis^1^** | 46.1 | 26.3 | | | 16.7 | | 10.9 | | |  |
| **Any Anxiety^1^** | 38.5 | 29.6 | | | 19.3 | | 12.6 | | |  |
| **Any Depressive^1^** | 31.8 | 36.4 | | | 13.6 | | 18.2 | | |  |
| **Any Behavioural^1^** | 26.3 | 35.6 | | | 23.1 | | 15.0 | | |  |
| **Any ADHD^1^** | 22.9 | 39.8 | | | 14.4 | | 22.9 | | |  |
| **Any less common disorder^1^** | 49.2 | 25.6 | | | 12.3 | | 12.8 | | |  |
| **Comorbidity^1^** | 17.9 | 41.0 | | | 19.4 | | 21.6 | | |  |

**Table S5: Model 4:** **latent class prior probabilities and sample prevalence for young people aged 11-16 years**

| **Indicators** | **Prevalence in sample** | **Class 1:**  ***No Services*** | **Class 2:**  ***Nonmedical Services*** | **Class 3:**  ***Community Services*** | **Class 4:**  ***Contact All Services*** |
| --- | --- | --- | --- | --- | --- |
|  | **%** | **%** | **%** | **%** | **%** |
| **Prior probability of membership** |  | 78.5 | 13.1 | 6.9 | 1.6 |
| **Item** | **Probabilities** | | | | |
| **Informal Help** | 13.3 | 0.04 | 0.34 | 0.57 | 0.89 |
| **School support** | 16.8 | <0.01 | 0.74 | 0.77 | 0.95 |
| **Mental Health Specialist** | 5.4 | <0.01 | 0.04 | 0.25 | 0.92 |
| **Primary Care** | 7.1 | <0.01 | <0.01 | 0.71 | 0.86 |
| **Educational support** | 5.1 | <0.01 | 0.10 | 0.24 | 1.0 |
| **Child Health** | 3.0 | <0.01 | <0.01 | 0.24 | 0.52 |

**Table S6: Proportions of class membership posterior probabilities by additional characteristics: young people aged 11-16 years**

| Covariate | | **Class 1**  ***No Services*** | | **Class 2**  ***Nonmedical Services*** | **Class 3**  ***Community Services*** | | **Class 4**  ***Contact all Services*** | | |  |
| --- | --- | --- | --- | --- | --- | --- | --- | --- | --- | --- |
|  | | **%** | | **%** | **%** | | **%** | | |  |
| ***Demographic characteristics*** | | | | | | | | | |  |
| **Tenure** | |  | |  |  | |  | | |  |
| *Own* | | 65.5 | | 64.7 | 58.8 | | 54.7 | | |  |
| *Private rented* | | 15.5 | | 16.0 | 13.4 | | 26.4 | | |  |
| *Social rented* | | 18.9 | | 19.3 | 27.8 | | 18.9 | | |  |
| **Income** | |  | |  |  | |  | | |  |
| *Highest tertile* | | 33.3 | | 30.0 | 25.3 | | 31.8 | | |  |
| *Middle tertile* | | 32.7 | | 35.5 | 38.6 | | 29.6 | | |  |
| *Lowest tertile* | | 34.0 | | 34.5 | 36.1 | | 38.6 | | |  |
| **Region^1^** | |  | |  |  | |  | | |  |
| *London* | | 12.2 | | 4.5 | 7.5 | | 5.7 | | |  |
| *South of England* | | 25.2 | | 35.7 | 26.7 | | 22.6 | | |  |
| *North of England* | | 32.2 | | 30.0 | 35.3 | | 41.5 | | |  |
| *Midlands and East of England* | | 30.5 | | 29.7 | 30.5 | | 30.2 | | |  |
| ***Within-household characteristics*** | | | | | | | | | |  |
|  | | | | **%** | **%** | **%** | | | **%** |  |
| **Parent Marital Status^1^** | | |  | |  |  | |  | | |
| *Married* | | | 67.5 | | 61.0 | 52.4 | | 41.5 | | |
| *Cohabiting* | | | 10.6 | | 13.2 | 9.1 | | 20.8 | | |
| *Lone parent – single* | | | 8.8 | | 12.3 | 19.3 | | 20.8 | | |
| *Lone Parent – previously married* | | | 13.2 | | 13.5 | 19.2 | | 17.1 | | |
| **Parental mental health^1^** | | |  | |  |  | |  | | |
| *Good* | | | 60.6 | | 48.6 | 39.9 | | 30.9 | | |
| *Fair* | | | 25.9 | | 29.3 | 31.2 | | 27.3 | | |
| *Poor* | | | 13.5 | | 22.1 | 29.0 | | 41.8 | | |
| **Problematic Family functioning^1^** | | | 16.6 | | 21.9 | 28.2 | | 34.7 | | |
| **YP is a carer** | | | 20.3 | | 20.5 | 18.8 | | 9.6 | | |
| ***Social characteristics*** | | | | | | | | | | |
|  | | **%** | | | **%** | **%** | **%** | | | |
| **Stressful life events^1^** | | | | |  |  |  | | | |
| *None* | | | 50.0 | | 31.7 | 24.2 | 19.6 | | | |
| *One* | | | 29.7 | | 30.8 | 30.7 | 35.3 | | | |
| *Two or more* | | | 20.4 | | 37.5 | 45.2 | 45.1 | | | |
| **Parent total difficulties scores^1^** | | | | |  |  |  | | | |
| *Close to average** | | | 89.5 | | 66.1 | 33.7 | 18.9 | | | |
| *Slightly raised* | | | 5.0 | | 10.8 | 13.9 | 11.3 | | | |
| *High* | | | 2.3 | | 5.4 | 15.5 | 11.3 | | | |
| *Very high* | | | 3.3 | | 17.7 | 36.9 | 58.5 | | | |
| *Close to average** | | | 89.5 | | 66.1 | 33.7 | 18.9 | | | |
| **Self reported total difficulties scores^1^** | | | | |  |  |  | | | |
| *Close to average** | | | 86.3 | | 67.7 | 48.1 | 35.7 | | | |
| *Slightly raised* | | 8.0 | | | 12.7 | 19.4 | 17.9 | | | |
| *High* | | 2.6 | | | 7.3 | 11.6 | 10.7 | | | |
| *Very high* | | 3.2 | | | 12.3 | 20.9 | 35.7 | | | |
| *Close to average** | | 86.3 | | | 67.7 | 48.1 | 35.7 | | | |
|  | |  | | |  |  |  | | | |
| **Broken friendship/ relationship^1^** | | 7.6 | | | 19.3 | 17.7 | 17.7 | | | |
| **Sent home for behaviour when at primary school^1^** | | 0.9 | | | 4.8 | 7.8 | 6.1 | | | |
| **YP’s general health^1^** | |  | | |  |  | |  | | |
| *Very Good / Good* | | 94.6 | | | 93.4 | 78.5 | | 67.9 | | |
| *Poor* | | 5.4 | | | 6.6 | 21.5 | | 32.1 | | |
| **Child with SEND^1^** | | 5.7 | | | 19.8 | 30.3 | | 50.0 | | |
| **Looked after by Social Services at any point^1^** | | 0.8 | | | 3.6 | 4.8 | | 7.6 | | |

**Table S7: Distribution of class membership posterior probabilities of characteristics for young people aged 11-16 years**

| Covariate | **Class 1**  ***No contact*** | | | **Class 2**  ***Nonmedical Services*** | | | **Class 3**  ***Community Services*** | | **Class 4**  ***Contact All Services*** | |  |  |  |
| --- | --- | --- | --- | --- | --- | --- | --- | --- | --- | --- | --- | --- | --- |
|  | **%** | | | **%** | | | **%** | | **%** | |  |  |  |
| ***Demographic characteristics*** | | | |  | | |  | |  | |  |  |  |
| **Gender** *Female* | 82.2 | | | 10.0 | | | 6.2 | | 1.7 | |  |  |  |
| *Male* | 80.8 | | | 11.6 | | | 5.8 | | 1.8 | |  |  |  |
| **Ethnicity** |  | | |  | | |  | |  | |  |  |  |
| *White/Other* | 78.5 | | | 12.2 | | | 7.2 | | 2.0 | |  |  |  |
| *Black/African/Caribbean* | 97.7 | | | 2.3 | | | 0.0 | | 0.0 | |  |  |  |
| *Asian/Asian British* | 94.1 | | | 4.0 | | | 2.0 | | 0.0 | |  |  |  |
| *Mixed/Multiple/Other* | 89.3 | | | 8.0 | | | 1.1 | | 1.6 | |  |  |  |
| **Tenure** |  | | |  | | |  | |  | |  |  |  |
| *Own* | 82.4 | | | 10.7 | | | 5.5 | | 1.4 | |  |  |  |
| *Private rented* | 81.0 | | | 11.0 | | | 5.2 | | 2.9 | |  |  |  |
| *Social rented* | 79.1 | | | 10.6 | | | 8.6 | | 1.7 | |  |  |  |
| **Income** |  | | |  | | |  | |  | |  |  |  |
| *Highest tertile* | 83.2 | | | 10.4 | | | 4.7 | | 1.8 | |  |  |  |
| *Middle tertile* | 79.5 | | | 11.9 | | | 7.0 | | 1.6 | |  |  |  |
| *Lowest tertile* | 80.4 | | | 11.3 | | | 6.3 | | 2.0 | |  |  |  |
| **Region** |  | | |  | | |  | |  | |  |  |  |
| *London* | 90.6 | | | 4.4 | | | 4.1 | | 0.9 | |  |  |  |
| *South of England* | 77.9 | | | 14.6 | | | 6.1 | | 1.5 | |  |  |  |
| *North of England* | 81.2 | | | 10.0 | | | 6.6 | | 2.2 | |  |  |  |
| *Midlands and East of England* | 81.7 | | | 10.5 | | | 6.1 | | 1.7 | |  |  |  |
| ***Within-household characteristics*** | | | | | | | | | | | |  |  |
|  | | ***%*** | ***%*** | | | | | ***%*** | ***%*** | | | |  |
| **Parent Marital Status** |  | | |  | | |  | |  | |  | | |
| *Married* | 84.1 | | | 10.0 | | | 4.8 | | 1.1 | |  |  |  |
| *Cohabiting* | 78.8 | | | 13.0 | | | 5.0 | | 3.2 | |  |  |  |
| *Lone parent – single* | 71.5 | | | 13.3 | | | 11.7 | | 3.6 | |  |  |  |
| *Lone Parent – previously married* | 78.7 | | | 10.6 | | | 8.5 | | 2.1 | |  |  |  |
| **Parental mental health** |  | | |  | | |  | |  | |  |  |  |
| *Good* | 85.7 | | | 9.2 | | | 4.2 | | 1.0 | |  |  |  |
| *Fair* | 79.2 | | | 11.9 | | | 7.0 | | 1.8 | |  |  |  |
| *Poor* | 69.3 | | | 15.0 | | | 10.9 | | 4.7 | |  |  |  |
| **Family functioning** |  | | |  | | |  | |  | |  |  |  |
| *Problematic* | 75.5 | | | 13.0 | | | 9.2 | | 3.3 | |  |  |  |
| **YP is a carer** | 82.3 | | | 11.0 | | | 5.7 | | 1.1 | |  |  |  |
| ***Social characteristics*** | | | | | | | | | | | | |  |
|  | **%** | | | | **%** | | **%** | | **%** | | | |  |
| **YP general health** |  | | |  | | |  | |  | |  | | |
| *Very Good / Good* | 82.3 | | | 10.7 | | | 5.0 | | 1.9 | |  |  |  |
| *Poor* | 62.3 | | | 10.0 | | | 18.2 | | 9.6 | |  |  |  |
| **YP with SEND** |  | | |  | | |  | |  | |  |  |  |
| **Looked after by Social Services at any point** | 45.7 | | | 26.1 | | | 19.6 | | 8.7 | |  |  |  |
| **Stressful life events** |  | | |  | | |  | |  | |  |  |  |
| *None* | 88.4 | | | 7.4 | | | 3.2 | | 1.1 | |  |  |  |
| *One* | 80.7 | | | 11.0 | | | 6.2 | | 2.1 | |  |  |  |
| *Two or more* | 68.8 | | | 16.7 | | | 11.3 | | 3.2 | |  |  |  |
| **Parent SDQ total difficulties scores** |  | | |  | | |  | |  | |  |  |  |
| *Close to average* | 88.2 | | | 8.6 | | | 2.5 | | 0.8 | |  |  |  |
| *Slightly raised* | 64.1 | | | 18.5 | | | 13.3 | | 4.1 | |  |  |  |
| *High* | 51.4 | | | 16.2 | | | 26.1 | | 6.3 | |  |  |  |
| *Very high* | 32.8 | | | 23.6 | | | 27.6 | | 16.0 | |  |  |  |
| **Self reported SDQ total difficulties scores** |  | | |  | | |  | |  | |  |  |  |
| *Close to average* | 88.1 | | | 8.3 | | | 2.9 | | 0.7 | |  |  |  |
| *Slightly raised* | 73.0 | | | 13.9 | | | 10.6 | | 2.5 | |  |  |  |
| *High* | 59.8 | | | 20.7 | | | 16.3 | | 3.3 | |  |  |  |
| *Very high* | 50.0 | | | 23.2 | | | 19.6 | | 7.3 | |  |  |  |
| **Broken friendship/relationship** | 64.4 | | | 21.5 | | | 11.1 | | 3.0 | |  |  |  |
| **Child sent home for behaviour from primary school** | 40.0 | | | 29.1 | | | 25.5 | | 5.5 | |  |  |  |
| **Experience bullying** |  | | |  | | |  | |  | |  |  |  |
| *Weekly or more* | 68.1 | | | 18.8 | | | 10.4 | | 2.8 | |  |  |  |
| *Monthly or less* | 77.1 | | | 14.7 | | | 6.5 | | 1.8 | |  |  |  |
| *Never* | 89.6 | | | 6.5 | | | 3.1 | | 0.9 | |  |  |  |
| **DSM-V Diagnoses** | | | | | | | | | |  |  |  |  |
|  | | | | | | | | | |  |  |  |  |
|  | **%** | | | | **%** | **%** | | | **%** |  |  |  |  |
| **Any DSM diagnosis** | 41.2 | | | 22.5 | | | 24.3 | | 12.0 | |  |  |  |
| **Any Anxiety** | 37.8 | | | 18.5 | | | 26.9 | | 16.9 | |  |  |  |
| **Any Depressive** | 36.4 | | | 23.2 | | | 22.2 | | 18.2 | |  |  |  |
| **Any Behavioural** | 29.5 | | | 22.4 | | | 33.3 | | 14.8 | |  |  |  |
| **Any ADHD** | 22.6 | | | 26.4 | | | 33.0 | | 17.9 | |  |  |  |
| **Any less common disorder** | 44.3 | | | 20.3 | | | 23.4 | | 12.0 | |  |  |  |
| **Comorbidity** | 27.9 | | | 21.6 | | | 31.4 | | 19.1 | |  |  |  |

**Table S8: Model 4: Latent class prior probabilities and sample prevalence by Support and Services for ages 17-19 years**

| **Indicators** | **Prevalence in sample** | **Class 1:**  ***No Services*** | **Class 2:**  ***Nonmedical Services*** | **Class 3:**  ***Specialised Services*** | **Class 4:**  ***Community & Specialised Services*** |
| --- | --- | --- | --- | --- | --- |
|  | **%** | **%** | **%** | **%** | **%** |
| **Prior probability of membership** |  | 71.1 | 17.6 | 7.6 | 3.7 |
| **Item** |  | **Probabilities** | | | |
| **Informal Help** | 39.7 | 0.22 | 0.85 | 0.76 | 0.96 |
| **School support** | 12.3 | <0.01 | 0.52 | <0.01 | 1.00 |
| **Mental Health Specialist** | 7.9 | <0.01 | 0.13 | 0.44 | 0.43 |
| **Primary Care** | 9.2 | <0.01 | <0.01 | 0.54 | 1.00 |
| **Educational support** | 2.7 | <0.01 | 0.12 | <0.01 | 0.18 |

**Table S9: Proportion of class membership posterior probabilities (%) of characteristics for young people aged 17-19 years**

| Covariate | | **Class 1**  ***No Services*** | | **Class 2**  ***Nonmedical Services*** | | **Class 3**  ***Specialised Services*** | | **Class 4**  ***Community & Specialised Services*** |  |
| --- | --- | --- | --- | --- | --- | --- | --- | --- | --- |
|  | | **%** | | **%** | | **%** | | **%** |  |
| ***Demographic characteristics*** | | | | | | | | |  |
| **Sexual Preference^1^** | |  | |  | |  | |  |  |
| *Heterosexual / Straight* | | 90.2 | | 83.3 | | 78.3 | | 65.7 |  |
| *Gay / Lesbian* | | 1.7 | | 3.6 | | 3.3 | | 11.4 |  |
| *Bisexual* | | 6.5 | | 8.3 | | 15.0 | | 20.0 |  |
| *Other sexual preference* | | 1.7 | | 4.8 | | 3.3 | | 2.9 |  |
| **Tenure** | |  | |  | |  | |  |  |
| *Own* | | 68.8 | | 63.4 | | 70.0 | | 55.3 |  |
| *Private rented* | | 13.5 | | 18.3 | | 10.0 | | 29.0 |  |
| *Social rented* | | 17.8 | | 18.3 | | 20.1 | | 15.8 |  |
| **Region** | |  | |  | |  | |  |  |
| *London* | | 11.2 | | 9.9 | | 8.1 | | 14.2 |  |
| *South of England* | | 25.6 | | 29.7 | | 29.0 | | 26.2 |  |
| *North of England* | | 31.2 | | 28.6 | | 32.3 | | 31.0 |  |
| *Midlands and East of England* | | 32.0 | | 31.9 | | 30.7 | | 28.6 |  |
| ***Within household characteristics* % % % %** | | | | | | | | |  |
| **Parent Marital Status** | |  | |  | |  | |  |  |
| *Married* | | 64.1 | | 67.0 | | 53.2 | | 59.5 |  |
| *Cohabiting* | | 10.5 | | 7.7 | | 14.5 | | 7.1 |  |
| *Lone parent – single* | | 12.0 | | 8.8 | | 16.1 | | 14.3 |  |
| *Lone Parent – previously married* | | 13.4 | | 16.5 | | 16.1 | | 19.1 |  |
| **Living with legal parent/guardian** | | 95.0 | | 96.7 | | 87.1 | | 92.9 |  |
| ***Social characteristics*** | | | | | | | | |  |
|  |  | | **%** | | **%** | | **% %** | |  |
|  |  | |  | |  | |  | |  |
| **Parental total difficulties scores^1^** | |  | |  | |  | |  |  |
| *Close to average* | | 93.9 | | 87.9 | | 77.4 | | 83.3 |  |
| *Slightly raised* | | 2.2 | | 3.3 | | 3.2 | | 4.8 |  |
| *High* | | 1.2 | | 5.5 | | 4.8 | | 4.8 |  |
| *Very high* | | 2.7 | | 3.3 | | 14.5 | | 7.1 |  |
| **Self total difficulties scores^1^** | |  | |  | |  | |  |  |
| *Close to average** | | 84.3 | | 65.1 | | 55.2 | | 52.6 |  |
| *Slightly raised* | | 7.5 | | 16.3 | | 27.5 | | 26.3 |  |
| *High* | | 3.4 | | 10.5 | | 3.5 | | 10.5 |  |
| *Very high* | | 4.8 | | 8.1 | | 13.8 | | 10.5 |  |
| *Close to average** | | 84.3 | | 65.1 | | 55.2 | | 52.6 |  |
|  | |  | |  | |  | |  |  |
| **Broken friendship/relationship^1^** | | 18.1 | | 29.0 | | 46.2 | | 26.7 |  |
| **YP general health^1^** | |  | |  | |  | |  |  |
| *Very Good / Good* | | 90.0 | | 76.9 | | 69.4 | | 71.4 |  |
| *Poor* | | 10.0 | | 23.1 | | 30.7 | | 28.6 |  |
| **Looked after by Social Services at any point** | | 1.1 | | 3.3 | | 3.2 | | 5.0 |  |
| **YP is a carer** | | 14.3 | | 17.6 | | 6.5 | | 17.5 |  |

**Table S10: Distribution of class membership posterior probabilities of characteristics for young people aged 17-19 years**

| Covariate | | **Class 1**  ***No Services*** | | **Class 2**  ***Nonmedical Services*** | **Class 3**  ***Specialised Services*** | | | **Class 4**  ***Community & Health Services*** |  |  |
| --- | --- | --- | --- | --- | --- | --- | --- | --- | --- | --- |
|  | | **%** | | **%** | **%** | | | **%** |  |  |
| ***Demographic characteristics*** | | | | | | | | |  |  |
| **Gender** *Female* | | 74.2 | | 11.4 | 8.5 | | | 5.9 |  |  |
| *Male* | | 83.9 | | 8.1 | 4.8 | | | 3.1 |  |  |
| **Ethnicity** | |  | |  |  | | |  |  |  |
| *White/Other* | | 79.0 | | 9.7 | 7.0 | | | 4.3 |  |  |
| *Black/Asian/ Mixed/Other* | | 79.9 | | 9.8 | 5.2 | | | 5.2 |  |  |
| **Sexual Preference** | |  | |  |  | | |  |  |  |
| *Heterosexual / Straight* | | 82.3 | | 8.9 | 6.0 | | | 2.9 |  |  |
| *Gay / Lesbian* | | 57.1 | | 14.3 | 9.5 | | | 19.1 |  |  |
| *Bisexual* | | 67.1 | | 10.0 | 12.9 | | | 10.0 |  |  |
| *Other sexual preference* | | 63.2 | | 21.1 | 10.5 | | | 5.3 |  |  |
| **Tenure** | |  | |  |  | | |  |  |  |
| *Own* | | 80.5 | | 8.8 | 7.1 | | | 3.6 |  |  |
| *Private rented* | | 74.4 | | 12.0 | 4.8 | | | 8.8 |  |  |
| *Social rented* | | 78.9 | | 9.6 | 7.7 | | | 3.9 |  |  |
| **Region** | |  | |  |  | | |  |  |  |
| *London* | | 80.58 | | 8.7 | 4.9 | | | 5.8 |  |  |
| *South of England* | | 77.2 | | 11.0 | 7.3 | | | 4.5 |  |  |
| *North of England* | | 79.7 | | 9.0 | 6.9 | | | 4.5 |  |  |
| *Midlands and East of England* | | 79.8 | | 9.8 | 6.4 | | | 4.0 |  |  |
| ***Within household characteristics*** | | | | | | | | |  |  |
|  | | **%** | **%** | | **%** | | | **%** |  | |
| **Parent Marital Status** | |  | |  |  | | |  |  |  |
| *Married* | | 80.0 | | 10.3 | 5.6 | | | 4.2 |  |  |
| *Cohabiting* | | 80.4 | | 7.2 | 9.3 | | | 3.1 |  |  |
| *Lone parent – single* | | 78.8 | | 7.1 | 8.9 | | | 5.3 |  |  |
| *Lone Parent – previously married* | | 75.0 | | 11.4 | 7.6 | | | 6.1 |  |  |
| **Living with legal parent/guardian** | | 79.6 | | 9.9 | 6.1 | | | 4.4 |  |  |
| **Current education/training/ employment** | |  | |  |  | | |  |  |  |
| *FT education (up to A Level/equivalent)* | | 74.6 | | 13.7 | 6.2 | | | 5.5 |  |  |
| *FT education (above A Level/equivalent)* | | 82.7 | | 9.7 | 4.1 | | | 3.6 |  |  |
| *Employment/apprenticeship* | | 85.6 | | 3.5 | 9.5 | | | 1.5 |  |  |
| *Not in education or training* | | 84.8 | | 3.8 | 8.8 | | | 2.5 |  |  |
| ***Social characteristics*** | | | | | | | | | |  |
|  | | ***%*** | | ***%*** | | ***%*** | ***%*** | | |  |
| **YP general health** | |  | |  |  | | |  |  |  |
| *Very Good / Good* | | 82.4 | | 8.6 | 5.3 | | | 3.7 |  |  |
| *Poor* | | 58.7 | | 16.7 | 15.1 | | | 9.5 |  |  |
| **YP has SEND** | | 72.5 | | 12.5 | 7.5 | | | 7.5 |  |  |
| **Looked after by Social Services at any point** | | 53.3 | | 20.0 | 13.3 | | | 13.3 |  |  |
| **YP is a carer** | | 79.7 | | 12.0 | 3.0 | | | 5.3 |  |  |
| **Parent total difficulties scores** | |  | |  |  | | |  |  |  |
| *Close to average* | | 81.0 | | 9.3 | 5.6 | | | 4.1 |  |  |
| *Slightly raised* | | 69.6 | | 13.0 | 8.7 | | | 8.7 |  |  |
| *High* | | 47.4 | | 26.3 | 15.8 | | | 10.5 |  |  |
| *Very high* | | 57.1 | | 8.6 | 25.7 | | | 8.6 |  |  |
| **Self total difficulties scores** | |  | |  |  | | |  |  |  |
| *Close to average* | | 84.6 | | 8.0 | 4.5 | | | 2.8 |  |  |
| *Slightly raised* | | 57.0 | | 15.1 | 17.2 | | | 10.8 |  |  |
| *High* | | 61.5 | | 23.1 | 5.1 | | | 10.3 |  |  |
| *Very high* | | 64.2 | | 13.2 | 15.1 | | | 7.6 |  |  |
| **Broken friendship/relationship^1^** | | 69.3 | | 12.5 | 13.6 | | | 4.6 |  |  |
| ***DSM-V Diagnoses*** | | | | | | | | | |  |
|  | **%** | | | **%** | **%** | | **%** | | |  |
| **Any DSM diagnosis** | | 54.8 | | 16.8 | 17.8 | | | 10.6 |  |  |
| **Any Anxiety** | | 53.7 | | 17.1 | 17.9 | | | 11.4 |  |  |
| **Any Depressive** | | 46.9 | | 12.2 | 24.5 | | | 16.3 |  |  |
| **Any Behavioural** | | 75.0 | | 12.5 | 12.5 | | | 0.0 |  |  |
| **Any ADHD** | | 64.3 | | 28.6 | 7.1 | | | 0.0 |  |  |
| **Any less common disorder** | | 53.7 | | 14.6 | 22.5 | | | 9.8 |  |  |
| **Comorbidity** | | 51.1 | | 13.3 | 22.2 | | | 13.3 |  |  |
